# Severe Guillain-Barré syndrome with concurrent optic neuritis in a pediatric patient: a case report

**DOI:** 10.3389/fimmu.2024.1517943

**Published:** 2025-01-17

**Authors:** Xin Xue, Yingcun Bao, Yakun Yu, Qun Li, Mei Liu, Xiaoling Li

**Affiliations:** ^1^ Department of Rehabilitation Medicine, Second Clinical College, Lanzhou University, Lanzhou University Second Hospital, Lanzhou, China; ^2^ Department of Rheumatology and Immunology, Second Clinical College, Lanzhou University, Lanzhou University Second Hospital, Lanzhou, China

**Keywords:** Guillain-Barré syndrome, optic neuritis, pediatric neurology, immunotherapy, rituximab, case report

## Abstract

**Introduction:**

Guillain-Barré syndrome (GBS) is a rare, immune-mediated polyneuropathy primarily affecting the peripheral nervous system. Diagnosis is often supported by neuroconduction studies showing polyradiculoneuropathy and cerebrospinal fluid (CSF) analysis revealing albumin-cytological dissociation. However, these features may not appear in the early stages, leading to potential misdiagnosis. The central nervous system (CNS) is generally not affected due to differences in antigen expression, optic neuritis (ON), a demyelinating inflammation of the optic nerve, occasionally co-occurs with GBS as a rare variant. Although GBS can manifest with various neurological symptoms, the co-occurrence of optic neuritis (ON) is rare, especially in pediatric cases. This report documents the first known case in China of a child with severe GBS complicated by ON, which developed following an upper respiratory infection.

**Case presentation:**

A 14-year-old male presented with acute progressive quadriparesis and visual impairment following a febrile illness. On admission, he displayed severe respiratory and autonomic instability requiring mechanical ventilation. Neurological examination revealed flaccid paralysis of all four limbs with absent reflexes, along with bilateral optic neuritis, confirmed by MRI showing inflammation of the optic nerve. Initial cerebrospinal fluid (CSF) analysis was normal, but subsequent testing revealed elevated protein levels typical of GBS. Neurophysiological studies indicated widespread demyelinating and axonal damage.

**Interventions and outcomes:**

The patient received intravenous immunoglobulin (IVIG) therapy, high-dose corticosteroids, and, given the severe progression, rituximab. Despite initial worsening, gradual improvement in muscle strength and visual acuity was observed over several weeks. At three months, the patient was discharged with significantly restored function, with muscle strength nearing baseline and partial visual recovery.

**Conclusion:**

This case highlights the clinical complexity of GBS with ON in pediatric patients, emphasizing the importance of timely immunomodulatory treatment. It also underscores the need for awareness of overlapping central and peripheral autoimmune neuropathies to improve diagnostic accuracy and patient outcomes.

## Introduction

Guillain-Barré Syndrome (GBS) is a rare, immune-mediated disorder characterized by rapid-onset, progressive weakness and areflexia, primarily affecting the peripheral nervous system (1). It is often preceded by an infection, most commonly viral or bacterial. Optic neuritis (ON), a demyelinating condition of the optic nerve, occasionally co-occurs with GBS, presenting an unusual challenge in diagnosis and treatment (2). The co-occurrence of these two conditions is extremely rare, particularly in pediatric populations.

This case report presents the first known pediatric case in China of GBS complicated by bilateral optic neuritis, triggered by an upper respiratory infection. The rarity of this combination, particularly in a child, underscores the complexity of diagnosis and treatment, highlighting the need for timely intervention to prevent severe sequelae. This report aims to contribute valuable insights into the pathophysiology, clinical management, and treatment outcomes of GBS/ON, with a particular focus on the role of rituximab, a drug not commonly used in GBS treatment.

## Case presentation

A 14-year-old male presented to the intensive care unit (ICU) at the Second Hospital of Lanzhou University with acute onset of progressive weakness in all four limbs over the preceding half-day. The patient had experienced a week-long upper respiratory infection with fever, for which he self-administered medications without significant improvement. On the night prior to admission, he developed sudden limb weakness with initial ambulation difficulties that did not improve with rest. By morning, he required assistance to walk, with symptoms progressing to include numbness in both feet, dysarthria, generalized muscle pain, and difficulty swallowing. As his condition continued to deteriorate, he was brought to the emergency department, where he experienced worsening weakness and difficulty breathing, requiring immediate ICU admission. Serological tests and PCR tests were done in the emergency room, but the results were negative.

## Initial examination and clinical findings

Upon admission, his vital signs included a body temperature of 36.5°C, respiratory rate of 29 breaths per minute, pulse of 111 beats per minute, and blood pressure of 128/76 mmHg. Respiratory examination revealed coarse breath sounds with wet rales in both lungs. Neurological examination showed preserved consciousness but reduced alertness, slurred speech, bilateral pupil dilation with prompt light reflexes, full range of eye movement without nystagmus, and decreased muscle tone in all four limbs. Proximal muscle strength was graded as 2/5 in both upper limbs and 1/5 in the lower limbs, with absent deep tendon reflexes and a negative Babinski sign bilaterally.

### Progression and imaging findings

**Figure 1:**
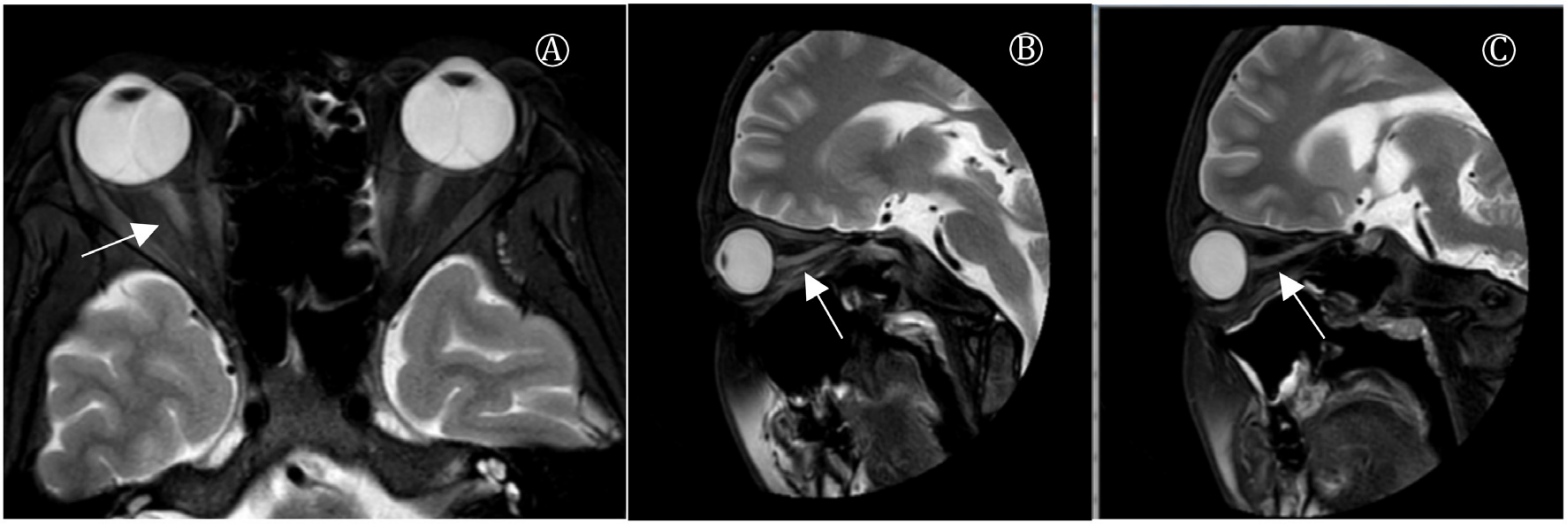
Plain MR scan of orbit. **(A)** Coronal image showed increased signal on T2WI in medial orbital and medial segments of bilateral optic nerve. **(B)** Sagittal image of left eye showed increased signal on optic nerve considering inflammatory changes. **(C)** Sagittal scan of the right eye indicated increased optic nerve signa.

After admission, the patient developed respiratory distress and hypoxemia (oxygen saturation fluctuating around 85%), necessitating endotracheal intubation and mechanical ventilation. On the second day, initial cerebrospinal fluid (CSF) analysis, serology for ganglioside and GQ1b antibodies, and anti-neuronal antibody testing were unremarkable. Cervical MRI also showed no abnormalities. Despite treatment, the patient’s condition rapidly progressed to total paralysis in all four limbs, with involvement of facial, respiratory, and swallowing muscles. The patient also reported visual blurring, with bilateral optic neuritis confirmed on orbital MRI, which revealed hyperintense signals in the intraorbital optic nerve segments (**Figure 1**).

### Neurological and laboratory assessments

GBS diagnosis relies on clinical examination, with classic diagnostic features including cerebrospinal fluid (CSF) protein-cell dissociation, abnormal electromyography (indicating sensory and/or motor conduction abnormalities typical of polyneuropathy; absent H reflex; increased distal motor latency or decreased compound muscle action potential (CMAP) amplitude in the facial nerve response). In this case, no characteristic changes in CSF were observed in the first week post-onset, with CSF protein elevation appearing only in the second week and repeat CSF analysis revealed a classic GBS finding of elevated protein without pleocytosis (**Table 1**).

**Table 1:** Laboratory results of lumbar puncture.

Test Date	Cerebrospinal Fluid Total Protein (g/L)	White Blood Cell Count (10*6/L)	Cerebrospinal fluid pressure(mmH_2_O)
2024-04-29	0.10 **↑**	2.00	140
2024-05-01	0.58 **↑**	8.00	120
2024-05-03	0.63 **↑**	4.00	90
2024-05-11	0.86 **↑**	2.00	100
2024-05-24	0.44 **↑**	0.00	110
2024-06-12	0.41 **↑**	3.00	120

The meaning of symbol ↑ is that the relevant indicators in the cerebrospinal fluid of the child after lumbar puncture are higher than normal.

Serum anti-myelin oligodendrocyte glycoprotein antibodies is 1:100 and Serum neurofascin-155 is negative. Electrophysiological studies showed absent sensory nerve action potentials in the median and ulnar nerves, reduced amplitude of motor evoked potentials (MEPs) in the ulnar, common peroneal, and tibial nerves, and slowed conduction velocities with prolonged latencies in both sensory and motor nerves. Needle electromyography indicated severe sensory and motor neuropathy with demyelinating and axonal injury (**Table 2**).

**Table 2:** Motor and sensory nerve conduction studies.

Determination of motor nerve function				
Type		Incubation period (MS)	Wave amplitude (mV)	Conduction velocity (m/s)
Ulnar nerve				
Left	carpus	5.86↑	1.79↓	16.2↓
	Above the elbow. - The wrist	21.3	0.062↓	
Right	carpus	3.33↑	1.52↓	13.1↓
	Above the elbow. - The wrist	24.0	0.16↓	
Median nerve				
Left	carpus	12.0↑	0.57↓	12.1↓
	Elbow-wrist	32.6	0.066↓	
Right	carpus	7.48↑	0.26↓	18.4↓
	Elbow-wrist	21.6	0.023↓	
Tibial nerve				
Left	anklebone	9.21↑	1.40↓	28.5↓
	Popliteal fossa - ankle	22.9	0.17↓	
Right	anklebone	7.80↑	0.73↓	25.5↓
	Popliteal fossa - ankle	22.3	0.092↓	
Common peroneal nerve				
Left	anklebone	13.2↑	0.065↓	26.0↓
	Fibula microcephalus - ankle	25.5	0.041↓	
Right	anklebone	8.56↑	0.54↓	33.2↓
	Fibula microcephalus - ankle	17.6	0.22↓	
Sensory nerve function measurement				
Type		Delay (MS)	Wave amplitude (μV)	Conduction velocity (m/s)
Median nerve				
Left	Finger III- wrist	Unelicited		
Right				

Multiple sensorimotor nerve lesions in extremities (severe demyelination with axonal injury); the conduction velocity and amplitude of bilateral motor nerve decreased, the incubation period was significantly prolonged, and the conduction velocity and amplitude of bilateral sensory nerve decreased.

↑ in column of Incubation period (MS) indicates that the motor nerve conduction time is prolonged and the nerve conduction speed is decreased.

↓ in Wave amplitude (mV) indicates that the amplitude of motor nerve potential is reduced and motion conduction is impaired.

## Therapeutic interventions

Following diagnosis, the patient was immediately admitted to the ICU and placed on mechanical ventilation. Intravenous immunoglobulin (IVIG) therapy was initiated at 0.4 g/kg/day for five consecutive days. Given the concurrent optic neuritis (ON), the clinical diagnosis was confirmed as Guillain-Barré syndrome with optic neuritis (GBS/ON). On the fourth day of IVIG treatment, high-dose methylprednisolone (500 mg/day for five days) was added, followed by tapering to 250 mg/day for five days and subsequently 120 mg/day for three days. The patient was then transitioned to oral methylprednisolone, initially dosed at 60 mg and tapered by 4 mg weekly, maintaining a low-dose regimen of 8 mg/day for long-term use.

Despite these interventions, the patient’s condition did not improve significantly after the initial course, with persistent quadriplegia and respiratory muscle weakness. Additionally, he developed a severe pulmonary infection, requiring tracheostomy and intensive antimicrobial therapy. Sixteen days post-IVIG, a trial of rituximab was administered given the absence of notable recovery, especially in motor strength. This led to a gradual improvement in muscle strength, with follow-up assessments showing strength gains in the upper limbs (proximal strength 2+, distal strength 1) and partial strength in the lower limbs.

**Figure 2:**
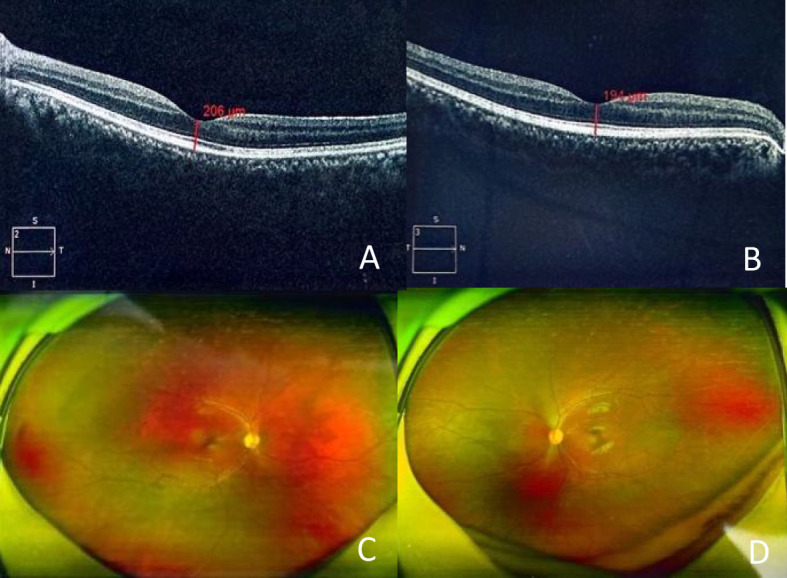
Optical coherence tomography OCT (**A** right eye, **B** left eye, thickened retinal nerve fiber layer around the optic papilla, unclear reflection in macular fovea), fundus photography FP (**C** right eye, **D** left eye, papillary edema, pale optic disc in both eyes).

Over the subsequent weeks, a second round of IVIG therapy (0.4 g/kg/day for five days) resulted in further functional improvement. The patient regained partial visual acuity in the right eye (50 cm finger-counting) and left eye (0.03 visual acuity), with color differentiation restored. On the 47th day of treatment, the patient was successfully weaned off mechanical ventilation, transitioning to autonomous breathing. After two months, he demonstrated significant recovery with muscle strength graded between II and III, visual acuity improved to 0.06 in the right eye and 0.04 in the left eye, visual evoked potentials showed a delay in P2 peak in both eyes, (**Table 3**).Optical coherence tomography (OCT) showed persistent retinal nerve fiber layer thickening and optic disc pallor (**Figure 2**). Enabling transfer from the ICU to the rehabilitation unit.

**Table 3:** Visual performance potential of the patient with Guillain-Barre syndrome complicated with optic neuritis.

Stimulus position	Lead	Incubation period (MS)		Wave amplitude (μV)	
		July 8, 2024	July 22, 2024	July 8, 2024	July 22, 2024
Left eye	N2	104.1	101.3	14.8	8.0
	P2	130.3	127.5		
Right eye	N2	113.4	109.7	12.0	7.2
	P2	136.9	139.7		

In rehabilitation, targeted therapies included physical exercise, physical agent modalities, hyperbaric oxygen, and nerve growth factor injections, along with vitamins B1 and B12. Visual function further improved as confirmed by ophthalmological assessments. After three months, the patient was discharged with muscle strength restored to grades IV and V, achieving near-independence in daily activities. At present, the patient has been ill for six months, and we have recently followed up and found that his motor function and color discrimination had fully recovered. But the level of vision has not fully recovered, the vision in the left eye was 6/24, and the vision in the right eye was 8/24. The patient was able to perform daily activities independently (**Table 4**).

**Table 4:** Symptoms and treatment timeline.

Date	Event	Notes
**One week before admission**	Fever, weakness, muscle aches	Thought it was a cold. Took ibuprofen and rested
**Admission Day**	Acute quadriparesis, respiratory distress, mechanical ventilation; initial CSF and imaging studies normal.	Rapid deterioration of condition.
**Day 2**	MRI confirmed bilateral optic neuritis.	Diagnosis of GBS/ON considered.
**Day 3**	IVIG initiated (0.4 g/kg/day for five days).	No significant improvement observed after IVIG.
**Day 8**	High-dose corticosteroids (500 mg/day methylprednisolone for five days, tapered subsequently).	Added due to lack of response to IVIG and presence of optic neuritis.
**Day 16**	Rituximab administered due to severe progression.	Gradual improvement in motor and visual function began.
**Day 20**	Repeat CSF and electrophysiology confirmed GBS with demyelination and axonal damage.	Diagnostic confirmation.
**Day 30**	Second course of IVIG administered.	Further improvement in strength and vision noted.
**Day 47**	Weaned off mechanical ventilation, transferred to rehabilitation.	Significant recovery in respiratory and motor function.
**Month 3**	Discharged with near-baseline motor function and partial visual recovery.	Follow-up confirmed sustained improvement.
**Month 6**	Motor function and color discrimination had fully recovered, the vision in the left eye was 6/24, and the vision in the right eye was 8/24.	Perform daily activities independently

The bold text indicates different days of disease course.

## Discussion

This case presents a rare co-occurrence of GBS and bilateral optic neuritis (GBS/ON), a combination that has been infrequently reported, especially in pediatric patients. While GBS is typically associated with peripheral nerve involvement, optic neuritis represents an uncommon, central nervous system manifestation. The pathophysiology of this dual involvement suggests a systemic autoimmune response that targets both central and peripheral myelin.

Our systematic review identified 33 prior GBS/ON cases. As of July 2024, 24 articles have been published worldwide, reporting cases of GBS combined with ON (3–27), with pediatric cases being particularly rare. Internationally, only three pediatric cases have been reported (4, 27) (**Table 5**). This case, involving a 14-year-old patient treated at the Second Hospital of Lanzhou University with GBS/ON, represents the first reported pediatric case in China. Including this case, there are a total of 34 cases, with a male-to-female ratio of 1:1. The majority of cases involve middle-aged adults, with a median onset age of 43 years (range: 7–72 years). Optic neuritis predominantly affects both eyes (24 out of 34 cases). Among the 34 cases, 12 exhibited simultaneous onset of GBS and ON, and 24% had respiratory muscle involvement.

**Table 5:** Data on three children previously reported.

Aspect	Patient 1 (12-year-old)	Patient 2 (14-year-old)	Patient 3 (7-year-old)
**Main Symptoms**	Bilateral optic neuritis (severe visual impairment), numbness in hands and feet	Optic neuritis (blurred vision), lower limb weakness, sensory abnormalities, autonomic dysfunction	Optic neuritis (right eye), quadriplegia, autonomic dysfunction, respiratory failure
**Neurological Type**	Demyelinating	Axonal (Acute Motor Axonal Neuropathy/AMAN evolving to AMSAN)	Axonal (AMAN variant)
**Antibody Findings**	Anti-Gal-C IgM positive, IgG negative, anti-aquaporin-4 antibody negative	Anti-ganglioside antibodies (GM1/GQ1b) negative	Anti-ganglioside antibodies (GM1/GQ1b) negative
**Imaging Results**	MRI showed optic nerve swelling; brain and spinal MRIs were normal	MRI showed optic nerve enhancement and cauda equina root enhancement	MRI showed nerve root enhancement; delayed appearance of optic nerve involvement
**Treatment**	Methylprednisolone pulse therapy (MPT)	Methylprednisolone, plasma exchange (PE), IVIG, and rituximab	IVIG, methylprednisolone, azithromycin
**Treatment Outcomes**	Full recovery of vision and resolution of numbness	Slow recovery over months; residual mild toe weakness	Full recovery with mild residual reduction in right eye visual acuity
**Special Notes**	First pediatric case of GBS and ON following Mycoplasma pneumoniae infection	Severe progression required mechanical ventilation and multiple immunotherapies	Autonomic dysfunction and delayed recognition of optic neuritis due to intubation and quadriplegia

The bold text indicates different aspects of clinical symptoms.

The pediatric case we report has the following novel aspects compared to the three previously reported pediatric cases: The patient rapidly developed quadriparesis and respiratory failure, requiring mechanical ventilation, with poor response to standard treatments (IVIG and corticosteroids).Rituximab was introduced during treatment, leading to gradual recovery of motor and visual functions, marking the first report of its use in GBS/ON. The simultaneous occurrence of GBS and optic neuritis (ON) suggests that the immune response may target both the peripheral and central nervous systems, highlighting potential immune cross-reaction. This is the first reported pediatric case in China of a 14-year-old male with co-occurring GBS and ON, offering new insights into the clinical management and treatment of this rare complication.

Previous reports have documented the coexistence of GBS with conditions like acute disseminated encephalomyelitis, transverse myelitis, multiple sclerosis, and ON (28). GBS combined with transverse myelitis is the most commonly reported syndrome, whereas GBS/ON is rarely reported (29). These cases are often associated with various infections, with most GBS cases having a history of antecedent infection, including cytomegalovirus (CMV), Epstein-Barr virus (EBV) (30), measles (31), and Mycoplasma pneumoniae, the most common antecedent pathogen in GBS (32). In one pediatric study, 7% of children with GBS had prior Mycoplasma pneumoniae infection (33). There have been reports suggesting that Mycoplasma pneumoniae can trigger isolated optic neuritis (34). Among GBS/ON cases, Mycoplasma pneumoniae is also the most common antecedent pathogen, though the causal relationship between Mycoplasma pneumoniae and autoimmune diseases remains unclear. Shared immune mechanisms, such as molecular mimicry, may have triggered concurrent demyelination in both the peripheral and central nervous systems. Additionally, whether GBS/ON represents an overlap syndrome or a continuous pathological process remains unclear (35). This patient had a history of antecedent infection with fever one week before onset. Unfortunately, pathogen testing was not performed before onset. Although serological and PCR testing for pathogens were negative, the preceding upper respiratory infection raises the possibility of molecular mimicry contributing to systemic autoimmunity. This mechanism may explain the simultaneous peripheral (GBS) and central (ON) nervous system involvement.

The sequential onset of quadriparesis and optic neuritis suggests a continuous pathogenesis involving shared autoimmune mechanisms targeting central and peripheral myelin. The absence of other CNS lesions on MRI ruled out conditions like multiple sclerosis and ADEM.

Visual impairment followed motor symptoms by one day, supporting the hypothesis of a systemic immune-mediated process. The absence of pain on eye movement suggests a non-ischemic inflammatory mechanism.

This case highlights the importance of co-existing GBS and ON in influencing treatment decisions. For GBS, plasma exchange (PE) or intravenous immunoglobulin (IVIG) is the primary treatment, while glucocorticoids may exacerbate muscle weakness due to steroid myopathy (36). However, glucocorticoids are also the main treatment for ON (37). Despite high-dose glucocorticoid and immunoglobulin therapy, the patient’s condition deteriorated rapidly to a state of quadriplegia and blindness, lasting approximately four weeks. Rituximab has been considered effective for central and peripheral nervous system diseases but has been associated with serious adverse events (38, 39).

There are no prior reports of Rituximab use in GBS/ON. This patient received multiple immunotherapies, including Rituximab, after no significant symptom relief was observed following initial IVIG and steroid treatment. The decision to initiate rituximab was based on the lack of response to initial treatments, and its introduction led to significant improvement in both motor strength and visual function, rituximab was chosen due to its targeted action on B cells, which play a central role in the pathogenesis of both GBS and optic neuritis. Alternatives like tocilizumab or oral immunosuppressants may be considered for future comparative studies. However, given the use of multiple immunosuppressants, the role of Rituximab in recovery remains uncertain. This pediatric case of GBS/ON highlights potential contradictions in clinical treatment. The complexity and severity of the condition posed significant treatment challenges, yet favorable outcomes were still achievable despite severe disease progression and prolonged course.

## Conclusion

This case underscores the complexity of managing Guillain-Barré syndrome with optic neuritis (GBS/ON), particularly in pediatric patients with severe presentations. The overlapping central and peripheral autoimmune manifestations posed unique diagnostic and therapeutic challenges, necessitating a multidisciplinary approach. Future research should focus on the underlying mechanisms linking GBS and ON, exploring whether these represent overlapping syndromes or distinct manifestations of systemic immune dysregulation. Establishing standardized treatment protocols for such rare cases is crucial to optimize outcomes and guide clinical practice.

## Data Availability

The original contributions presented in the study are included in the article/**Supplementary Material**. Further inquiries can be directed to the first author.
