# Supplementary material for: Severe Guillain-Barré syndrome with concurrent optic neuritis in a pediatric patient: a case report
Source: Front Immunol. 2025 Jan 17;15:1517943. doi: 10.3389/fimmu.2024.1517943 (PMC11781990; doi:10.3389/fimmu.2024.1517943)
Supplement: Supplementary file 1 [file DataSheet1.pdf]

兰大二院肌电图诱发电位报告

检查号: 2024-6-0148 | 检查日期: 2024-06-19 | 出生日期: 2009-09-26 | 病历号: 1399171
患者姓名: 王皓 | 性别: 男性 | 年龄: 14 | 身高:

Sensory Nerve Conduction Studies

SNCS Nerve	Peak Lat ms	Amp uV	CV m/s	Dist mm
尺神经 感觉 右				
指 V - 腕	2.00	15.2	55.0	110
正中神经 感觉 左				
指 III - 腕	2.77	13.9	46.9 ↓	130
正中神经 感觉 右				
指 III - 腕	2.63	16.5	45.6 ↓	120
腓浅神经 感觉 左				
外踝 - 足背外侧	1.90	16.7	44.7	85.0
腓浅神经 感觉 右				
外踝 - 足背外侧	1.90	11.6	44.7	85.0
腓肠神经 感觉 左				
踝 - sura	2.31	11.2	43.3	100
腓肠神经 感觉 右				
踝 - sura	1.92	9.4	46.9	90.0

Motor Nerve Conduction Studies

MNCS Nerve	Lat ms	Amp mV	CV m/s	Dist mm
尺神经 运动 左				
腕 - ADM	**3.72** ↑	**2.3** ↓		70.0
肘上-腕	13.1	1.23 ↓	26.7 ↓	250
尺神经 运动 右				
腕 - ADM	**3.75** ↑	4.3 ↓		70.0
肘上-腕	12.3	1.99 ↓	30.4 ↓	260
正中神经 运动 左				
腕 - APB	**6.89** ↑	0.34 ↓		70.0
肘-腕	14.7	0.47 ↓	**29.4** ↓	230
正中神经 运动 右				
腕 - APB	**6.66** ↑	0.27 ↓		70.0
肘-腕	14.7	0.42 ↓	**27.4** ↓	220
胫神经 运动 左				
踝 - AH	7.00 ↑	3.7 ↓		80.0
腘窝-踝	16.5	2.0 ↓	38.9 ↓	370
胫神经 运动 右				
踝 - AH	5.15 ↑	3.2 ↓		85.0
腘窝-踝	18.1	1.81 ↓	28.6 ↓	370

MNCS				
腓总神经 运动 左				
踝 - EDB	8.08 ↑	0.47 ↓		80.0
腓骨小头-踝	17.2	0.28 ↓	30.7 ↓	280
腓总神经 运动 右				
踝 - EDB	5.55 ↑	0.81 ↓		80.0
腓骨小头-踝	13.6	0.70 ↓	38.5 ↓	310

F-Wave

右 尺神经 FResponse					
	F-M Lat	CV	M-Lat	#F	
	ms	m/s	ms	#	F%
腕 - ADM	52.1 ↑	--	4.2	5.0	100

右 胫神经 FResponse					
	F-M Lat	CV	M-Lat	#F	
	ms	m/s	ms	#	F%
踝 - AH	67.2 ↑	--	6.1	4.0	80.0

H-Reflex

左 胫神经 HReflex			
	M-Lat	H-Lat	H/M Amp
	ms	ms	-
腘窝 - 腓肠肌	8.9	74.2 ↑	--

右 胫神经 HReflex			
	M-Lat	H-Lat	H/M Amp
	ms	ms	-
腘窝 - 腓肠肌	8.2	未引出	--

EMG MUP Data

	插入延长	静纤颤	止正相	束颤	轻收缩时限 MS	运动单位波幅 UV	电位多相%	重收缩运动波型	电位波幅 MV
右 胫前肌	——	1+	1+	——	正常		增多	单混相	
右 腓肠肌	——	1+	1+	——	正常		增多	单混相	
右 肱二头肌	——	——	——	——	正常				
右 骨间肌 I	延长	2+	3+	——	正常		增多	单纯相	

1 双正中神经感觉神经传导速度减慢，波幅正常。
2 右尺神经、双腓肠神经、双腓浅神经感觉神经传导均正常。
3 双尺神经、双正中神经、双胫神经、双腓总神经运动神经传导速度减慢，波幅降低且波形离散，远端潜伏期均延长。
4 右尺神经、右胫神经 F 波传导潜伏期延长。
5 右胫神经 H 反射未引出；左胫神经 H 反射潜伏期延长。
6 右骨间肌 I 、右胫前肌、右腓肠肌可见神经源性损害改变，余被检肌尚可。
提示：肌电图示四肢多发感觉运动神经损害复查，较 2024-5-16 结果明显改善（潜伏期缩短；波幅增高，速度增快），请结合临床。

检查者：石玉军　　　审核者：
