# Supplementary figures and images for: Severe Guillain-Barré syndrome with concurrent optic neuritis in a pediatric patient: a case report
Source: Front Immunol. 2025 Jan 17;15:1517943. doi: 10.3389/fimmu.2024.1517943 (PMC11781990; doi:10.3389/fimmu.2024.1517943)

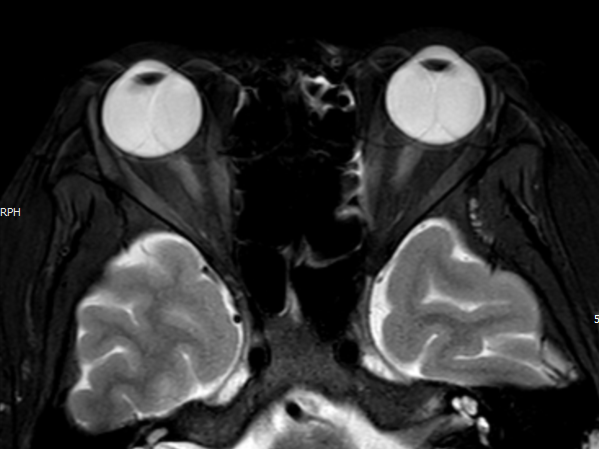

Supplement: Supplementary file 2 [file Image1.png]

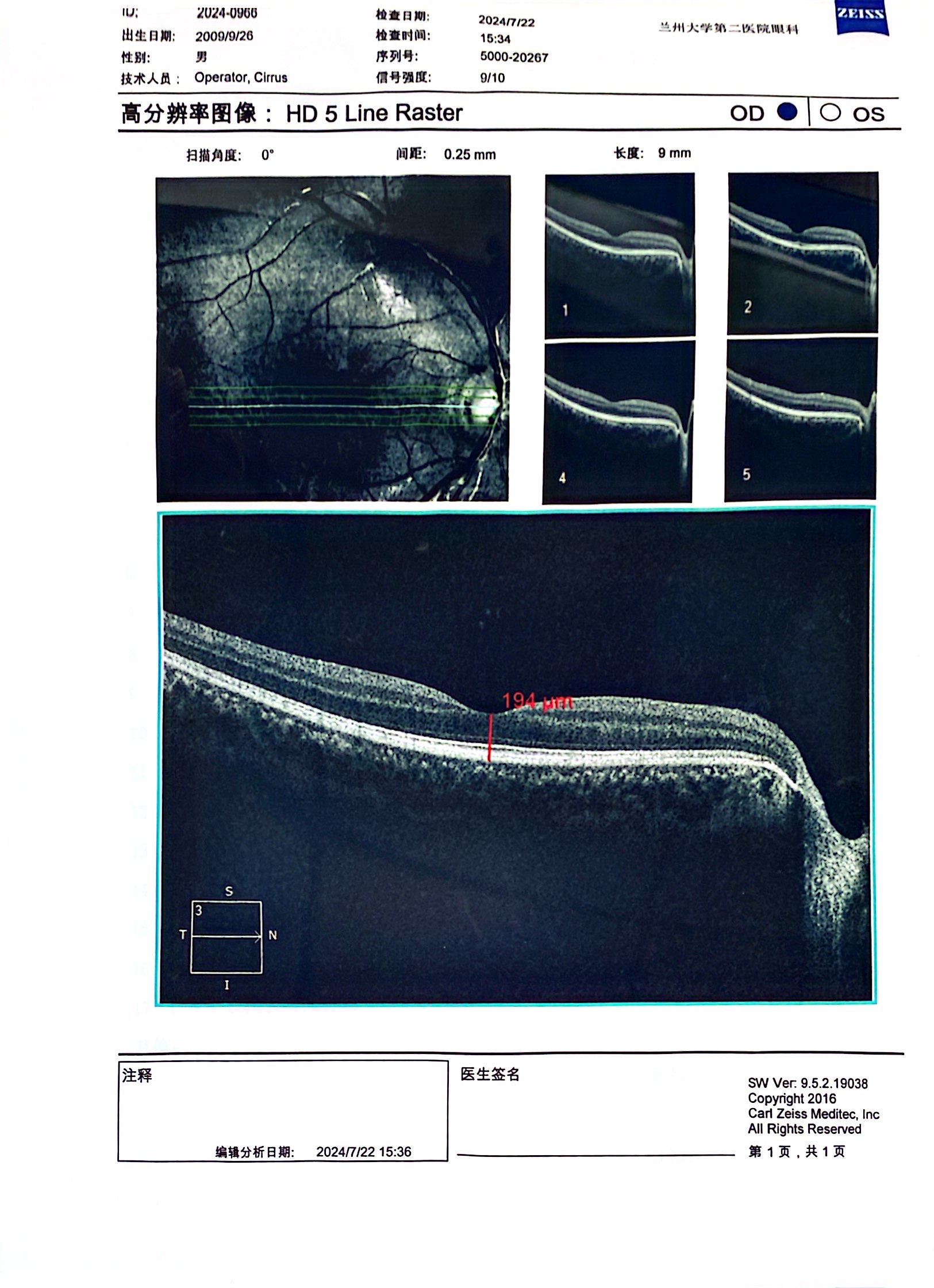

Supplement: Supplementary file 3 [file Image2.jpeg]

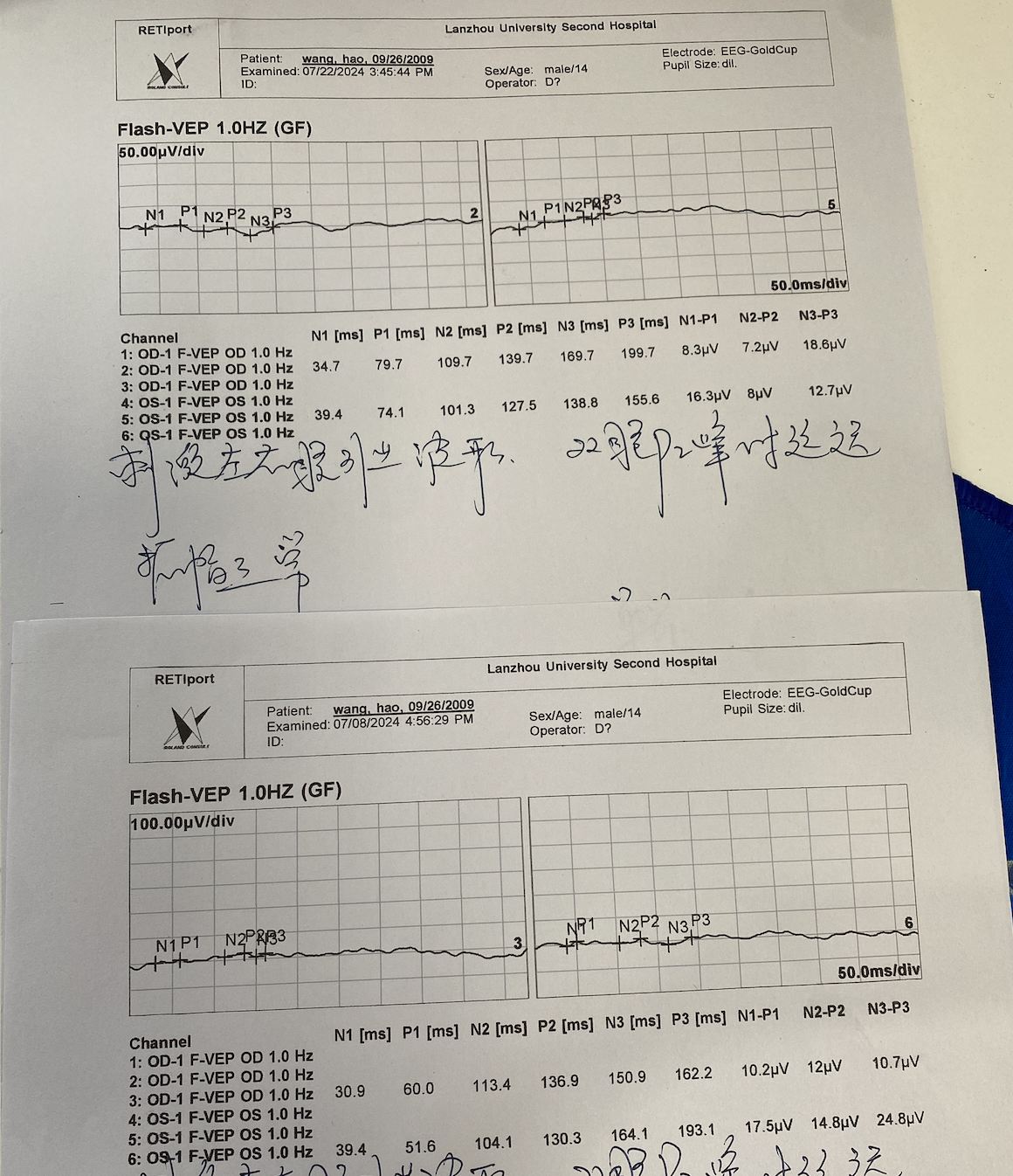

Supplement: Supplementary file 4 [file Image3.png]
